# Rapid Microfluidic Immuno-Biosensor Detection System for the Point-of-Care Determination of High-Sensitivity Urinary C-Reactive Protein

**DOI:** 10.3390/bios14060283

**Published:** 2024-05-30

**Authors:** Szu-Jui Chen, Song-Yu Lu, Chin-Chung Tseng, Kuan-Hsun Huang, To-Lin Chen, Lung-Ming Fu

**Affiliations:** 1 Department of Engineering Science, National Cheng Kung University, Tainan 70101, Taiwan; n98091063@gs.ncku.edu.tw (S.-J.C.); n98091055@gs.ncku.edu.tw (S.-Y.L.); n96104496@gs.ncku.edu.tw (K.-H.H.); n98134049@gs.ncku.edu.tw (T.-L.C.); 2 Division of Nephrology, Department of Internal Medicine, National Cheng Kung University Hospital, Tainan 70101, Taiwan; chinchun@mail.ncku.edu.tw; 3 College of Medicine, National Cheng Kung University, Tainan 70101, Taiwan

**Keywords:** microfluidic, microchip, ELISA, CKD, C-reactive protein

## Abstract

A microfluidic immuno-biosensor detection system consisting of a microfluidic spectrum chip and a micro-spectrometer detection device is presented for the rapid point-of-care (POC) detection and quantification of high-sensitivity C-reactive protein (hs-CRP) in urine. The detection process utilizes a highly specific enzyme-linked immunosorbent assay (ELISA) method, in which capture antibodies and detection antibodies are pre-deposited on the substrate of the microchip and used to form an immune complex with the target antigen. Horseradish peroxidase (HRP) is added as a marker enzyme, followed by a colorimetric reaction using 3,3′,5,5′-tetramethylbenzidine (TMB). The absorbance values (a.u.) of the colorimetric reaction compounds are measured using a micro-spectrometer device and used to measure the corresponding hs-CRP concentration according to the pre-established calibration curve. It is shown that the hs-CRP concentration can be determined within 50 min. In addition, the system achieves recovery rates of 93.8–106.2% in blind water samples and 94.5–104.6% in artificial urine. The results showed that the CRP detection results of 41 urine samples from patients with chronic kidney disease (CKD) were highly consistent with the conventional homogeneous particle-enhanced turbidimetric immunoassay (PETIA) method’s detection results (R^2^ = 0.9910). The experimental results showed its applicability in the detection of CRP in both urine and serum. Overall, the results indicate that the current microfluidic ELISA detection system provides an accurate and reliable method for monitoring the hs-CRP concentration in point-of-care applications.

## 1. Introduction

C-reactive protein (CRP) is a key marker of inflammation and can increase up to 1000-fold at sites of infection or inflammation [1,2]. It is thus commonly monitored in patients undergoing hemodialysis or peritoneal dialysis, since during acute inflammation, the liver produces CRP as an acute-phase reactant to coordinate the inflammatory reaction [3]. Recent investigations have revealed that CRP is also present in urine, although at lower concentrations than in blood. Thus, it is regarded as a valuable biomarker for chronic kidney disease (CKD), urinary tract infections, pyelonephritis, glomerulonephritis, and other inflammatory conditions of the urinary system [4]. Urine collection is non-invasive, easily accessible, and repeatable, making it an attractive option for monitoring disease progression and guiding treatment decisions [5]. Consequently, monitoring the CRP concentration in urine is of significant interest for the early detection of infections and inflammatory responses.

The most commonly used techniques for CRP detection in clinical laboratories include enzyme-linked immunosorbent assays (ELISAs) [6,7,8], immunoturbidimetric assays [9,10,11], latex immunoagglutination assays [12,13], fluorescence assays [14,15,16], chemiluminescence assays [17,18], surface plasmon resonance-based assays [19], and electrochemical assays [20,21,22,23,24,25]. However, these methods are time-consuming and require skilled operators. Moreover, they require bulky and expensive precision instrumentation. Consequently, they are infeasible for ongoing patient management in point-of-care testing (POCT) [26,27,28,29,30] and home healthcare contexts, where frequent monitoring is desirable.

Commercial ELISA methods are considered to be one of the most effective and powerful techniques for the detection and quantification of proteins, antibodies, hormones, and pathogens in biological samples [31]. Of the various ELISA methods available, the sandwich ELISA method, in which the antibody of interest is captured through ionic and hydrophobic interactions, is one of the most commonly employed approaches for CRP detection due to its greater sensitivity than other ELISA methods such as immunonephelometric detection, turbid immunometric detection, enzyme-linked immunosorbent detection, optical plasmonic immunoassays detection, and fluorescence detection with immunoreaction. [32]. Nonetheless, benchtop ELISA methods are time-consuming and require high reagent volumes. Consequently, there is a need for automated and miniaturized immunoassay platforms capable of reducing the time and cost of the ELISA process [33,34,35].

Microfluidic detection technology provides many advantages for biomedical applications due to its high integration capability and excellent biocompatibility [36,37,38,39,40,41]. As the microfluidics field has advanced, many microfluidic lab-on-a-chip (LOC) devices have been developed that can achieve sample injection, separation, mixing, coating, transmission, and detection on a single platform [42,43,44,45,46,47,48]. LOC-based immunoassays have thus emerged as a powerful tool for biomedical research and clinical diagnostics [49,50,51,52,53]. By integrating multiple laboratory functions into a single microchip, LOC-based immunoassays offer significant advantages over traditional laboratory-based immunoassays, including superior portability, miniaturization, high throughput, sensitivity, rapid analysis, automation, and cost-effectiveness. These advantages have prompted the development of a wide range of LOC-based immunoassays for the detection of proteins, nucleic acids, and small molecules, with applications ranging from early-stage diagnosis to ongoing disease monitoring and treatment [54,55,56,57,58]. To address the issues of high reagent volume consumption and time-consuming ELISA processes, there is a need for the development of an automated and miniaturized immunoassay platform.

**Figure 1:**
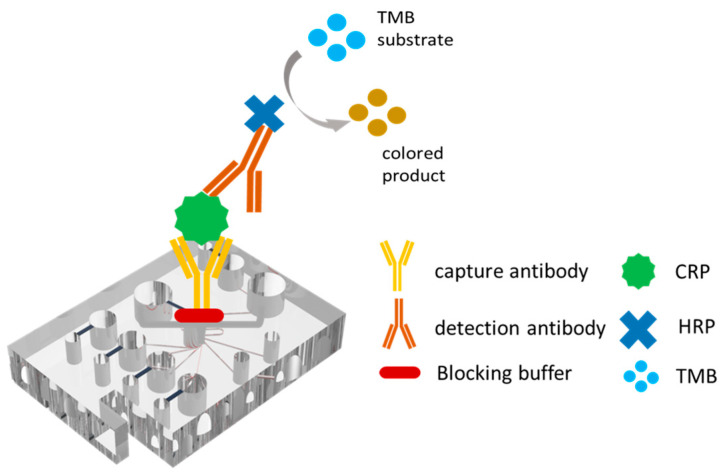
ELISA detection principle for determination of CRP concentration.

This study presents a microfluidic ELISA detection system to determine the CRP concentration in urine samples. As shown in Figure 1, capture and detection antibodies are pre-deposited on a substrate to bind the target antigen, forming an immune complex. Horseradish peroxidase (HRP) is introduced as a labeling enzyme, and the resulting compound is processed with 3,3′,5,5′-tetramethylbenzidine (TMB) to initiate a color reaction. Finally, the absorbance of the reacted sample is measured using a micro-spectrometer detection device to quantify the hs-CRP concentration. The microfluidic spectrum chip serves as a low-cost and effective replacement for the 96-well plate used in traditional spectral measurements. Results show that the current system provides the ability to perform rapid hs-CRP measurements with a minimal detection liquid volume, fast reaction time, high sensitivity, and low cost.

## 2. Materials and Methods

### 2.1. Reagents

The reagents used in this study included a DuoSet Ancillary Reagent Kit 2 (DY008), a DuoSet ELISA Human C-Reactive Protein/CRP kit (DY1707), reagent diluent concentrate 2 (DY995), ELISA plate-coating buffer (DY006), and Quantikine ELISA wash buffer 1 (WA126). All the reagents were purchased from R&D Systems (Minneapolis, MN, USA). The reagent kits contained a capture antibody (Polyclonal Anti-C-Reactive Protein Antibody) and hs-CRP antigen detection antibody (Mouse Anti-Human CRP Capture Antibody), TMB (3,3′,5,5′-tetramethylbenzidine), Streptavidin-HRP (horseradish peroxidase), wash buffer (Tween 20 in PBS), stop solution (2N, H_2_SO_4_), PBS (2.7 mM KCl, 137 mM NaCl, 1.5 mM KH_2_PO_4_, 8.1 mM Na_2_HPO_4_, pH 7.2–7.4), and reagent diluent (1% BSA in PBS, pH 7.2–7.4). The artificial urine was purchased from Analab INC. (1700-0017, Taipei, Taiwan). It has a pH value of 6.6 and a composition close to real human urine, and it is in a sterile state.

### 2.2. Manufacturing and Assembly of Microfluidic Spectrum-Chip

**Figure 2:**
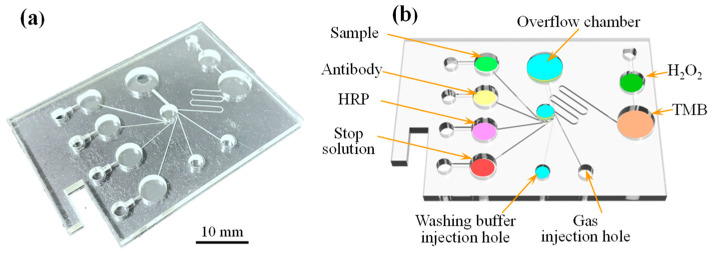
(**a**) Photograph of spectrum-microchip and (**b**) schematic illustration showing main components of spectrum-microchip.

Figure 2 shows the complete structure of the microfluidic spectrum-chip proposed in this study, comprising the main PMMA chip for reagent storage, injection, overflow, mixing, sample reaction and detection, and a PET cover layer. The chip and cover layers had thicknesses of 3 mm and 0.1 mm, respectively, and were patterned using a CO_2_ laser processing machine (14W, Giant Technology Co., Taipei, Taiwan). The PMMA chip contained six reagent tanks (Reagent A, sample, ϕ 7 mm; Reagent B, detection antibody, ϕ 7 mm; Reagent C, HRP, ϕ 7 mm; Reagent D, stop solution, ϕ 7 mm; Reagent E, H_2_O_2_, ϕ 5 mm; and Reagent F, TMB, ϕ 7 mm); seven sample injection zones (ϕ 7 mm); an overflow buffer zone (ϕ 10 mm); a detection zone (ϕ 5 mm); and several reagent tanks connected to the detection area through microchannels with widths and depths of 180 μm and 90 μm, respectively. The PET cover layer had the form of a simple rectangular slide with dimensions of 70 × 50 mm^2^. After the PMMA microchip was patterned (also with a size of 70 × 50 mm^2^), the two substrates were bonded under a maximum pressure of 40 kg/cm^2^ for 15 s to complete the chip assembly.

### 2.3. Micro-Spectrometer Detection Device

**Figure 3:**
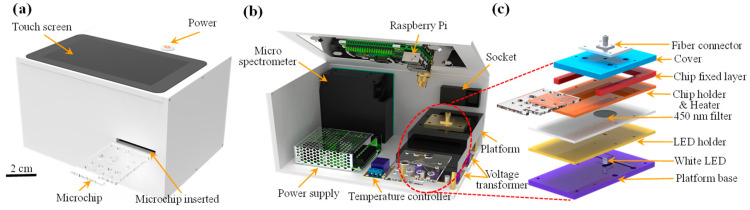
Schematic illustrations showing: (**a**,**b**) main components of the detection system and (**c**) detailed components of the detection system and placement of the microfluidic chip.

Figure 3a,b show the precision micro-spectrometer detection box developed in this study with dimensions of 200 × 150 × 120 mm^3^ and a weight of approximately 900 g. The main components of the detection box included a micro spectrometer (SE2020-025-VNIR, SmartEngine, Hsinchu, Taiwan), a relay (KY-019, SONGLE, Taipei, Taiwan), a Raspberry Pi touch tablet computer (Raspberry Pi 3 Model B, Raspberry Pi Foundation, Tokyo, Japan), a temperature controller, a power switch module (JR-101-1FR, RLEIL, New Taipei City, Taiwan), a buck/boost converter module (DC-DC, 4A-XL6009E1, XLSMIL, Shanghai, China), and a power supply (5V, RS-15-5, Meanwell Co., New Taipei City, Taiwan).

Figure 3c show the detailed structure and components of the microfluidic detection platform. To detect the intensity of the light passing through the reaction area, such that the concentration of the hs-CRP could be determined, a micro-spectrometer and white light source were positioned above and below the reaction area of the chip, respectively, once it was inserted into the detection platform. The white light was filtered through a central filter with a wavelength of 450 nm, passed through the reaction area, and transmitted to the micro-spectrometer through a fiber optic cable connected to an aluminum alloy base with an SMA (SubMiniature version A, Fusoh Shoji Co., Tokyo, Japan) connector.

### 2.4. Experimental Details

We prepared a capture antibody stock solution at a concentration of 360 μg/mL by mixing lyophilized protein powder with 1 mL of PBS buffer and further diluted the solution with PBS buffer to acquire a final concentration of 2 μg/mL. A standard solution of CRP antigen with a concentration of 120 μg/L was prepared by dissolving 500 μL of lyophilized powder in PBS buffer containing 1% BSA. Serial dilutions were then performed to obtain control samples with CRP antigen concentrations of 0.1, 1, 10, 50, 150, 300, 500, 800, 1000, 1200, and 1500 ng/mL. A stock solution of detection antibody was prepared by dissolving 45 μg of lyophilized protein powder containing 1% BSA in 1 mL of PBS buffer. The solution was further diluted using PBS buffer containing 1% BSA to acquire a final concentration of 0.25 μg/mL. A working solution of HRP was obtained by diluting stock HRP solution with PBS solution to acquire a final concentration that was 200-fold lower than that of the original solution. Finally, equal volumes of color reagents A (containing H_2_O_2_) and B (containing TMB) were mixed to prepare the ELISA color substrate solution. The mixture was incubated for 15 min before use, allowing the TMB to be oxidized by the H_2_O_2_ and form a blue color.

The capture antibody was added to the reaction area of the microchip. The reaction area was covered with a sealing film to prevent evaporation, and the microchip was then incubated in a shade environment at 20 °C for 10 h. After the incubation period, a washing solution was used to remove any unbound capture antibodies from the reaction area. A BSA-blocking solution was added to the detection area, and the microchip was incubated at 25 °C for 1 h. Following incubation, the reaction area was once again flushed with a washing solution to remove any unbound BSA. As shown in Figure 2, all reagents except the CRP sample were added to each chamber in the microchip and sealed for use when testing was required.

In the detection process, the CRP test sample was introduced into the chip and driven to the central reaction chamber using a syringe. The detection antibody and HRP label solution were then serially added to the reaction chamber under syringe pressure. Once the sample reaction was complete, washing buffer was used to remove any unbound detection antibody and HRP from the chamber. Subsequently, H_2_O_2_ was injected into the chip and directed into the TMB chamber. The resulting mixture exited the TMB chamber and flowed through a serpentine microchannel to achieve uniform mixing. Finally, the mixture entered the central reaction area for color development. Upon completion of the color development process, the entire chip was inserted into the micro-spectrometer detection device for the measurement of hs-CRP concentration.

## 3. Results and Discussion

### 3.1. Optimal Reaction Temperature on Microfluidic Spectrum-Chip

**Figure 4:**
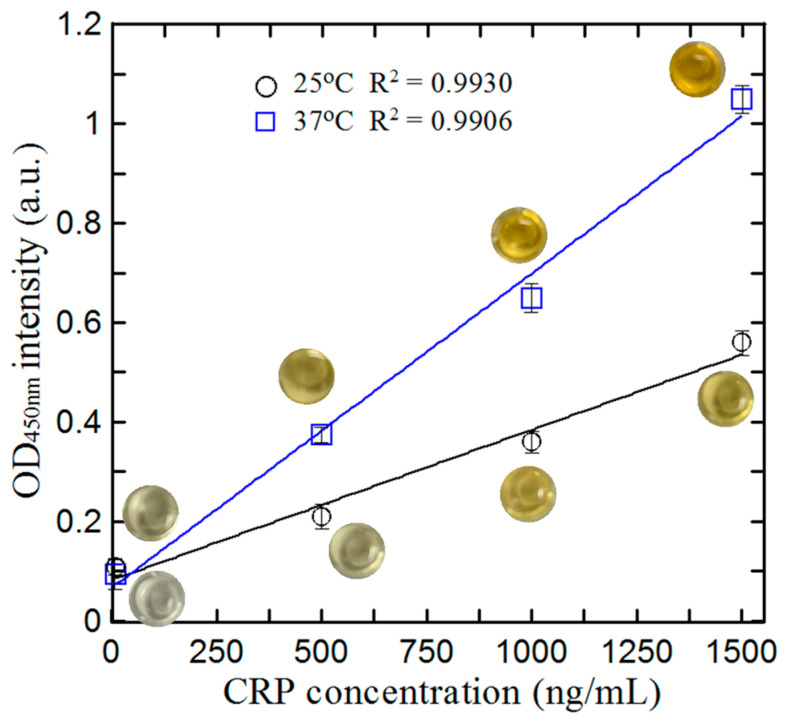
Effect of antibody–antigen reaction temperature on absorbance value for different CRP concentrations.

The optimal temperature for the protein immunobinding stage of the reaction process was ascertained by measuring the absorbance of the reaction product formed at two different temperatures (25 °C and 37 °C) using a light source with a wavelength of 450 nm. The results are presented in Figure 4 for hs-CRP concentrations ranging from 1 ng/mL to 1500 ng/mL. The correlation coefficient (R^2^) of the detected intensity signal is greater than 0.99 for both temperatures. However, the slope of the curve obtained for a reaction temperature of 37 °C is greater than that of the reaction product formed at 25 °C. In other words, for a given concentration range, the absorbance values at 37 °C have a wider range (maximum absorbance value − minimum absorbance value) than those at 25 °C. In general, a standard curve with a broader range of absorbance values indicates a better sensitivity and enables more efficient experimental observations. Thus, based on the results presented in Figure 4, the temperature for the reaction process was set to 37 °C in all the remaining experiments.

### 3.2. Optimal Reagent/Reaction Time on Microfluidic Spectrum-Chip

**Figure 5:**
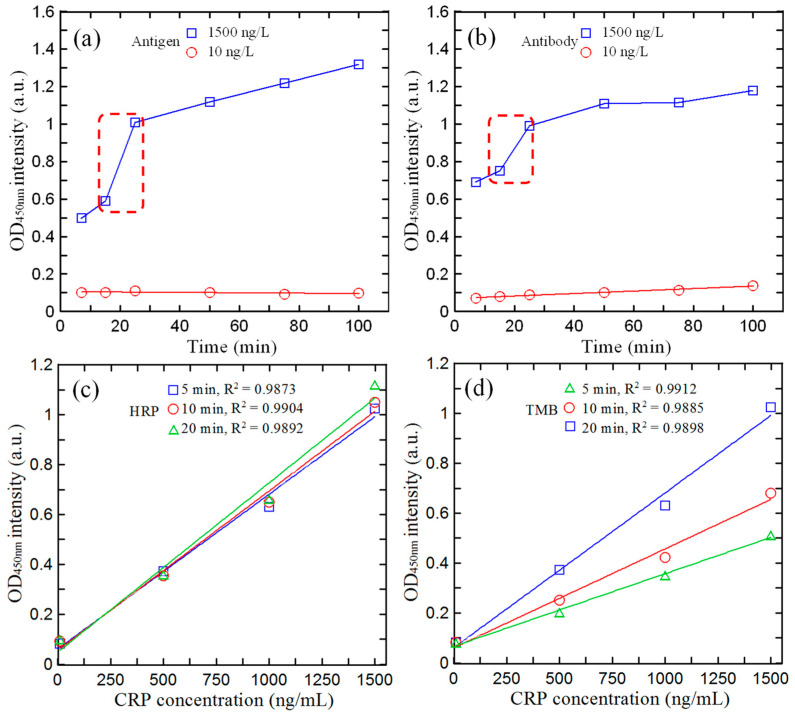
Effects of reaction time on absorbance value for different reagent and CRP concentrations: (**a**) antigen, (**b**) antibody, (**c**) HRP, and (**d**) TMB.

The optimal reaction time for antigen, antibody, HRP, and TMB detected by hs-CRP on a microfluidic chip was determined through a series of experiments. Figure 5a shows the absorbance values obtained after different reaction times in the interval of 5–100 min for antigen concentrations of 10 and 1500 ng/mL. Figure 5b shows the corresponding results for the antibody. For a concentration of 10 ng/mL, the absorbance has a very low value of approximately 0.1 absorbance units (a.u.) regardless of the reaction time for both reagents. However, at a high concentration of 1500 ng/mL, the absorbance increases rapidly as the reaction time increases to 25 min, and then it gradually levels off in both cases. In other words, the optimal binding between the CRP and functional groups occurred after 25 min, and the reaction time was set accordingly in all the subsequent experiments.

Figure 5c presents the variation in the absorbance intensity with the hs-CRP concentration given antigen and antibody reaction times of 25 min, a reaction temperature of 37 °C, and HRP reaction times of 5, 10, and 20 min. For all three reaction times, the values of the correlation coefficient (R^2^) were greater than 0.985, indicating a good linearity between the absorbance and the reaction time. Hence, for efficiency reasons, the HRP reaction time was set to the lowest value of 5 min (R^2^ = 0.9873). Figure 5d shows the variation in the absorbance with the hs-CRP concentration for TMB reaction times of 5, 10, and 20 min. In this case, the longest reaction time of 20 min results in the highest sensitivity and second highest correlation coefficient (R^2^ = 0.9898). A reaction time of 20 min was thus used in all the remaining experiments.

### 3.3. Effect of Detection Path Length on Absorbance Intensity

**Figure 6:**
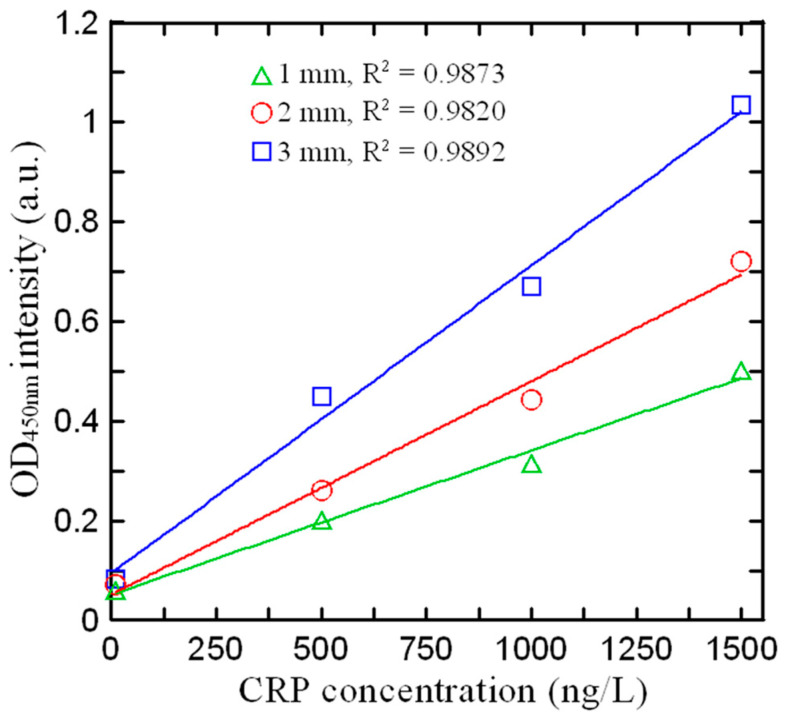
Effects of spectrum-microchip thickness on absorbance value for different CRP concentrations.

According to the Beer–Lambert law, the absorbance of a liquid is positively related to the detection optical path length. Thus, to optimize the detection performance, the path length (i.e., the thickness of the PMMA chip in the current case) must be appropriately designed. Three initial sample volumes were identified through a binary search algorithm, namely 150 μL, 100 μL, and 50 μL, corresponding to detection path lengths of 3.0 mm, 2.0 mm, and 1.0 mm, respectively. Figure 6 shows the intensity values obtained for four control samples with known CRP concentrations in microchips with PMMA layer thicknesses of 1.0 mm, 2.0 mm, and 3.0 mm, respectively. For all three detection path lengths, the correlation coefficient (R^2^) exceeds 0.98. However, the detection path length of 3 mm results in the steepest slope, indicating the highest detection sensitivity. This finding is consistent with the Beer–Lambert law. Although longer detection path lengths can further improve the detection performance of microchips, this conflicts with the goal of minimizing the detection platform size. Thus, in fabricating the chip for further experiments, the PMMA layer thickness was set to 3 mm.

### 3.4. Calibration of Microfluidic ELISA Detection System

**Figure 7:**
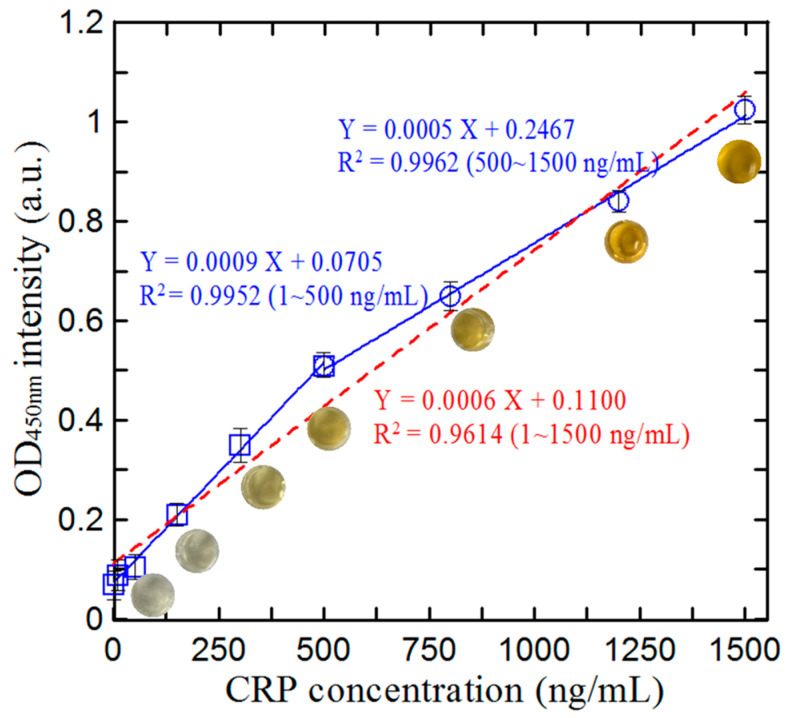
Absorbance value measurements for artificial urine samples with known CRP concentrations ranging from 1 to 1500 ng/mL.

To calibrate the current microfluidic ELISA detection system, detection experiments were performed under the optimal conditions described above to detect the spectral intensity values of nine standard artificial urine CRP samples with known concentrations ranging from 1 to 1500 ng/mL. For each sample, five spectral intensity measurements were acquired and the values were averaged to determine a representative measurement. Figure 7 shows the corresponding results. The red dotted line shows the fitting results obtained over the full concentration range. The correlation coefficient (R^2^) has a low value of 0.9614. Consequently, the corresponding regression relationship (Y = 0.0006 X + 0.1100) has only a limited predictive ability for the CRP concentration. Regression analysis was separately applied to concentration ranges of 1–500 ng/mL and 500–1500 ng/mL. Each measured concentration intensity (Y) was substituted into the regression equation Y = 0.0006 X + 0.1100. By evaluating the error between the calculated intensity and the measured intensity, the delimitation point between the two concentration ranges was determined. The results indicated that the largest error occurred at a CRP concentration of 500 ng/mL. Therefore, the delimitation point between the concentrations of the two CRP ranges was set at 500 ng/mL. A more detailed analysis revealed that the variation in the absorbance with the CRP concentration comprised two distinct regions of 1–500 ng/mL and 500–1500 ng/mL. The corresponding regression equations were found to be Y = 0.0009 X + 0.0705 and Y = 0.0005 X + 0.2467, with correlation coefficients of R^2^ = 0.9952 and 0.9962, respectively. Both correlation coefficients are close to 1, indicating the validity of each equation for predicting the CRP concentration in the corresponding ranges. Moreover, the limit of detection (LOD) of the microfluidic ELISA detection system is 0.1 ng/mL.

### 3.5. Detection of CRP Concentration in Blind Samples

**Figure 8:**
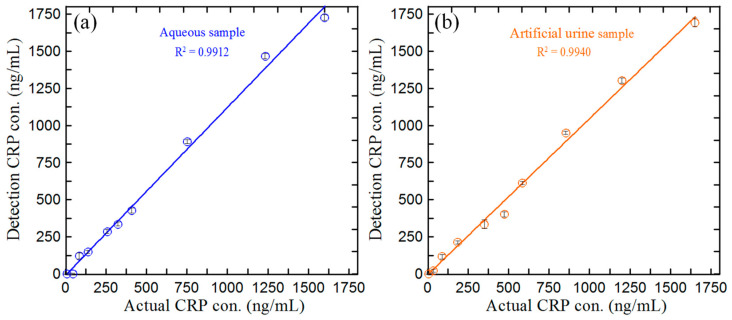
Comparison of CRP detection results and actual CRP concentration measurements in blind sample tests: (**a**) aqueous samples and (**b**) artificial urine samples.

The feasibility of the current microfluidic ELISA detection system for practical CRP detection was investigated using 20 random blind samples (10 aqueous samples and 10 artificial urine samples) with CRP concentrations between 0 and 1700 ng/mL. The CRP concentration of each sample was detected using the current microfluidic ELISA detection system; the optimal reaction conditions are shown in Section 3.1, Section 3.2 and Section 3.3 and the calibration equation is shown in Section 3.4. Figure 8a,b compare the CRP measurements obtained using the proposed device for the aqueous and urine samples, respectively, with the actual CRP concentrations of the samples. Note that the plotted values are the average values obtained over three repeated experiments in both cases. There is good agreement between the two sets of measurements for the two samples (i.e., R^2^ = 0.9912 for the aqueous samples and R^2^ = 0.9940 for the artificial urine samples). Moreover, the sample recoveries ranged from 93.8~106.2% and 94.5~104.6% for the aqueous and artificial urine samples, respectively. Thus, the accuracy of the proposed microfluidic ELISA detection system is confirmed.

### 3.6. Application of Proposed Microfluidic ELISA Detection System to hs-CRP Determination in Real-World Urine Samples

**Figure 9:**
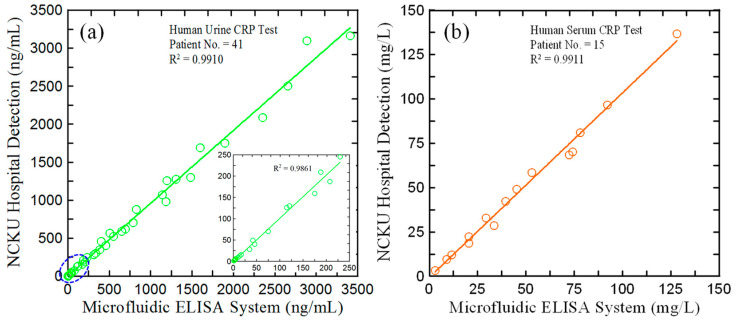
Comparison of CRP detection results obtained by the conventional homogeneous PETIA method (*y*-axis) and the proposed microfluidic system (*x*-axis) for (**a**) 41 real-world urine samples and (**b**) 15 real-world serum samples.

The accuracy and feasibility of the current microfluidic ELISA detection system was further evaluated by measuring the hs-CRP concentration in urine samples of 41 adult CKD patient volunteers (from National Cheng Kung University Hospital (NCKUH) in Taiwan). Participants participating in the study provided fresh urine samples in accordance with NCKUH IRB protocol # IRB-A-ER-108-527, and written consent was obtained. Each sample is assigned a unique identifier based on the donor, date of collection, and method of collection. Participants’ personal details were not recorded. The urine samples were pretreated for testing by the hospital before delivery, and their CRP concentrations were measured using a homogeneous PETIA method conducted on a benchtop biochemical analyzer (Architect c16000, Abbott Co., Abbott Park, IL, USA). Figure 9a compares the detection results obtained using the current microfluidic ELISA detection system with those obtained using the conventional benchtop system. Note that the CRP concentrations of the 41 urinary samples collected from NCKUH ranged from 200 ng/dL to 3500 ng/dL. In order to evaluate the detection performance of the microfluidic ELISA detection system at lower CRP concentrations (i.e., 1~200 ng/dL), 20 original samples were diluted 100 and 200 times with deionized (DI) water. The results showed that good agreement was observed between the two sets of measurements (R^2^ = 0.9910). In addition, this microfluidic ELISA detection system can also be applied to the detection of CRP in human serum. Figure 9b shows the CRP concentration results of serum samples of 15 adult CKD patient volunteers obtained using the homogeneous PETIA detection method and the proposed microfluidic detection system, respectively. As shown, the correlation coefficient between the two sets of measurements is 0.9911. Table 1 presents a qualitative comparison of the microfluidic ELISA detection system with several previous methods reported in the literature for the detection of CRP concentration in human serum or urine samples. Therefore, the feasibility of the current microfluidic ELISA detection system for practical applications of urinary hs-CRP concentration determination was confirmed.

## 4. Conclusions

This study has presented a microfluidic ELISA chip and micro-spectrometer detection device for the determination of hs-CRP in urine samples. The experimental results have shown that the optimal detection performance is obtained at a reaction temperature of 37 °C using reaction times of 25, 5, and 20 min for the antigen–antibody binding reaction, HRP labeling reaction, and TMB color development reaction, respectively. In addition, the optimal chip thickness has been shown to be 3 mm. Two standard calibration curves have been presented for extracting the CRP concentration from the measured absorbance values over the ranges of 1–500 ng/mL and 500–1500 ng/mL. The corresponding correlation coefficients are 0.9952 and 0.9962, respectively. Moreover, the LOD and recovery rate of the proposed system are 0.1 ng/mL and 93.8%~106.2%, respectively. The practical utility of the proposed system for real-world hs-CRP measurement applications has been demonstrated by comparing the measurement results obtained for 41 real-world CKD urine samples with those obtained using a homogeneous PETIA method. It has been shown that the two sets of results are in excellent agreement (R^2^ = 0.9910). Overall, the results confirm that the current microfluidic ELISA detection system provides a simple, fast, reliable, and accurate method for the POC monitoring of urine hs-CRP concentrations in clinical and home healthcare settings.

## Figures and Tables

**Table 1:** Comparison of analytical methods in CRP assay.

Method	Material	Analysis Time	Device Price	Sample Type	Detection Range	LOD	Ref.
ELISA	citicoline-BSA conjugate, AuNPs-aptamer nanozyme	>3 h	High	Rat blood	0.1–200 ng/mL	8 pg/mL	[6]
Immuno-turbidimetric	BSA, PEG-6000-CRP conjugate	45 min	High	Human serum	1–500 ng/mL	0.54 ng/mL	[9]
LFIA	CRP capture antibody, polyclonal goat anti-mouse IgG	30 min	High	Human serum	0.1–500 ng/mL	0.1 ng/mL	[12]
Fluorescent	Goat antibody to human CRP	--	High	Serum	0.4–52 mg/L	0.4 mg/L	[14]
Chemiluminescence	Monoclonal mouse anti-CRP antibodies, Poly-HRP streptavidin	30 min	High	Human serum	10–100 ng/mL	0.49 ng/mL	[17]
Electrochemical	Anti-CRP antibody	--	High	Human serum	0.1–1000 ng/mL	1 pg/mL	[24]
Metal-enhanced chemiluminescence	Ab1 and detection antibodies (Ab2) of CRP	60 min	High	Human serum	0.7–7000 ng/mL	0.05 ng/mL	[59]
Lab-on-Chip immunoassays	guided-mode resonance sensor intergrated microfluidic chip, CRP antibody	>2 h	Low	Rat blood	0.64–5000 ng/mL	3.2 ng/mL	[60]
Lab-on-Chip electrochemical	P3-CRP peptide	60 min	High	Human serum	0.01~10^5^ ng/mL	47 pg/mL	[61]
Proposed method	Polyclonal Anti-CRP Antibody, Mouse Anti-Human CRP Capture Antibody	<50 min	Low	Human serum and urine	1–1500 ng/mL	0.1 ng/mL	This work

## Data Availability

The data presented in this study are available upon request from the corresponding author.
